# Busulfan and cyclosphamide induce liver inflammation through NLRP3 activation in mice after hematopoietic stem cell transplantation

**DOI:** 10.1038/srep17828

**Published:** 2015-12-04

**Authors:** Jianlin Qiao, Yujin Huang, Yuan Xia, Peipei Chu, Haina Yao, Linyan Xu, Kunming Qi, Yun Liu, Kailin Xu, Lingyu Zeng

**Affiliations:** 1 Department of Hematology, the Affiliated Hospital of Xuzhou Medical College, Xuzhou 221002, China; 2 Blood Diseases Institute, Xuzhou Medical College, Xuzhou 221002, China; 3 Key Laboratory of Bone Marrow Stem Cell, Jiangsu Province, Xuzhou 221002, China

## Abstract

The aim of this study was to evaluate the role of NLRP3 inflammasome on BU/CY-induced liver inflammation in mice after HSCT. HSCT mice model was established through infusion of 5 × 10^6^ bone marrow mononuclear cells after conditioned with BU/CY. On day 7, 14, 21 and 28 after HSCT, mice were sacrificed for analysis of liver inflammation, cytokine secretion, NLRP3 expression and caspase-1 activation as well as release of ATP and high-mobility group protein B1 (HMGB1). Furthermore, NLRP3 selective inhibitor (BAY 11-7082) was administrated into mice after HSCT to evaluate its effects on liver inflammation. Severe liver inflammation and damage with elevated secretion of IL-1β and IL-18 were found in mice after HSCT. Meanwhile, elevated expressions of NLRP3 and caspase-1 activation in liver were found. In addition, increased release of ATP and HMGB1 were observed. Selective inhibition of NLRP3 decreased caspase-1 activation and secretion of IL-1β and IL-18. Furthermore, NLRP3 inhibition also reduced infiltration of macrophages and neutrophils and improved liver function. In conclusion, NLRP3 was involved in BU/CY-induced liver inflammation after HSCT and selectively inhibited it ameliorated liver inflammation and improved liver function, suggesting targeting NLRP3 might be a new approach in the prophylaxis of liver inflammation after HSCT.

Hematopoietic stem cell transplantation (HSCT) is currently considered as an effective therapy for patients with hematological malignancies, including leukemia and multiple myeloma^1^,^2^. However, severe complications, such as hepatic veno-occlusive disease (HVOD), graft-versus-host disease (GVHD), were observed in patients receiving HSCT, severely limiting the treatment efficiency and outcomes, leading to higher mortality^2^,^3^,^4^. HVOD, also known as sinusoidal obstruction syndrome, is a condition which is characterized by the obstruction of small veins in the liver and usually develops within 30 days after HSCT with a highly variable incidence ranging from 10 to 60%^5^,^6^.

The pathogenesis of HVOD is very complicated with lots of factors involved. There is increasing evidence demonstrating that damage of liver sinusoidal endothelial cells (LSECs) in zone 3 of the hepatic acinus was the main initiating factor for the development of HVOD^7^,^8^. Our previous studies showed that preconditioning treatment employed before HSCT, either total body irradiation or busulfan combined with cyclosphamide (BU/CY), caused damage to LSECs leading to loss of normal structural integrity of liver sinusoid, abnormal liver function and subsequent occurrence of HVOD^9^. In addition, inflammatory cell infiltrations into liver were also observed, suggesting inflammation might be involved in the liver injury after HSCT.

Inflammasomes are multiprotein complexes that respond to various inflammatory stimuli through mediating the activation of caspase-1, which promotes the secretion of pro-inflammatory cytokines, IL-1β and IL-18^10^,^11^. NLRP3 inflammasome is one of the largest as well as the most studied cytosolic inflammasome, which is comprised of the NOD-like receptor NLRP3 (containing the NLR-typical elements and an N-terminal PYD), the adapter protein termed apoptosis-associated speck-like protein containing a caspase recruitment domain (ASC) and procaspase-1^12^. The exact mechanism of NLRP3 inflammasome assembly and subsequent activation is still not very clear and is believed that upon inflammatory stimulation, NLRP3 undergoes oligomerized, leading to recruitment of ASC through interaction of PYD, which activates pro-caspase-1 via interaction of an caspase recruitment domain (CARD) located within N-terminal of ASC^13^,^14^. Activated caspase-1 enzyme is formed through the heterodimerization of two p20 and two p10 molecules, which are generated from autocatalytic cleavage of pro-caspase-1^15^,^16^. Activated caspase-1 cleaves pro-IL-1β and pro-IL-18 into mature form, IL-1β and IL-18. Mature IL-1β is predominantly produced by macrophages, neutrophils and monocytes and reported to be involved in the regulation of the activation of lymphocytes and endothelial cells^17^, whereas, mature IL-18 can induce IFN-γ and several pro-inflammatory cytokines secretion as well as activate NK cells.

Our previous study showed more severe liver inflammation, damage and higher occurrence of HVOD were observed in mice after BU/CY + HSCT than TBI + HSCT^9^. BU/CY is widely used as a conditioning approach in clinic and investigating the mechanism of liver inflammatory injury induced by BU/CY in recipients would have significantly clinical importance. Given the regulation of inflammation of NLRP3, we aimed to evaluate its role in liver inflammation in mice after BU/CY + HSCT and showed NLRP3 inflammasome was associated with liver inflammation and subsequent liver damage. Furthermore, selective inhibition of NLRP3 function ameliorated liver inflammation and improved liver function, suggesting therapeutically targeting NLRP3 inflammasome might be beneficial in the prophylaxis of liver injury after HSCT.

## Results

### Liver inflammation after HSCT

**Figure 1:**
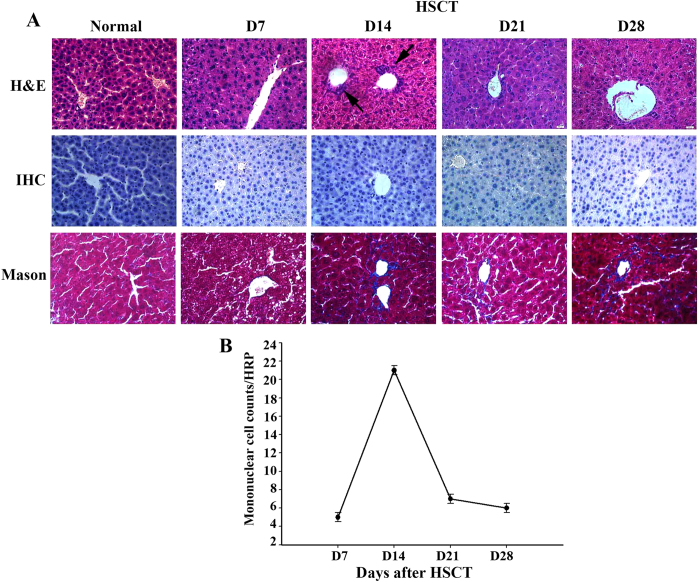
Liver pathological changes and numbers of mononuclear cells in liver post HSCT. At indicated time points after HSCT, mice were sacrificed and liver was isolated and fixed with formaldehyde, dehydrated, waxed and sliced into 4 μm thickness by RM2126 microtome for H&E staining (**A**). For immunohistochemical staining, the slices were incubated with 3% H_2_O_2_ at room temperature and blocked with 5–10% goat serum. Then the slices were incubated with pan-endothelial cell monoclonal antibody (MECA-32) at 37 °C for 1–2 h followed by the incubation with secondary antibody. Color was developed with 3,3′-diaminobenzidine. Mason staining was performed using a commercial kit. A representative staining graph selected from 6 independent stainings in each group was shown. Meanwhile, numbers of infiltrated mononuclear cells in liver were also counted (**B**).

The normal liver lobule is a hexagonal mass of tissue primarily composed of plates of hepatocytes radiating from the region of the central vein toward the periphery. Consistent with our previous study^9^,^18^, H&E staining of liver after HSCT showed abnormal structure of liver lobule, edema and coagulative necrosis of hepatocytes, liver sinusoid obstruction, narrowing and obstruction of central vein and liver sinusoid as well as inflammatory cells infiltration (Fig. 1A). Following studies revealed increased mononuclear cells counts were observed in liver post HSCT, reaching a maximum on day 14 (Fig. 1B). Immunohistochemical staining by using endothelial cell monoclonal antibody (MECA-32) indicated loss of integrity of sinusoid wall, abnormal structure of liver lobule and LSECs detachment. In addition, fibrin deposition was also observed in central vein and liver sinusoid as demonstrated by mason staining. According to Deleve scoring system for evaluating liver damage (Table 1), increased liver damage was observed from d5 post HSCT and reached a severest level on d14, followed by a moderate amelioration on d20, consistent with the changes of mononuclear cells in liver (Fig. 1B), demonstrating the contribution of inflammation to liver damage.

### Increased secretion of IL-1β and IL-18

**Figure 2:**
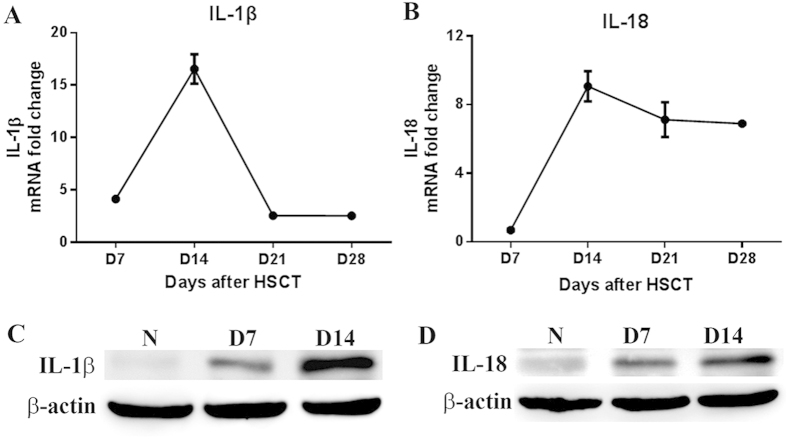
Expression of IL-1β and IL-18 in liver post HSCT. On d7, d14, d21 and d28 post HSCT, RNA was extracted from liver for analysis of mRNA expression of IL-1β (**A**) and IL-18 (**B**) by quantitative real-time PCR. Meanwhile, protein expressions of IL-1β (**C**) and IL-18 (**D**) on d7 and d14 post HSCT were also measured by western blot.

IL-1β and IL-18, two powerful pro-inflammatory cytokines with pleiotropic activities, are demonstrated to play a critical role in the regulation of inflammation. To evaluate their role in liver inflammation, RNA was extracted from liver at indicated time points after HSCT, which was used to measure the mRNA level. As seen in Fig. 2A,B, mRNA expression of IL-1β and IL-18 increased from d7 post HSCT and achieved a maximum on d14, followed by a decrease on d21, which were consistent with the temporal changes of liver inflammation as well as liver damage as demonstrated by scoring system. Consistent with mRNA expression profiles, protein expression of IL-1β and IL-18 increased from d7 to d14 after transplantation (Fig. 2C,D).Taken together, pro-inflammatory cytokines IL-1β and IL-18 secreted from infiltrated inflammatory cells might be responsible for liver inflammation and subsequent liver damage.

### Elevated activation of caspase-1 and expression of NLRP3

**Figure 3:**
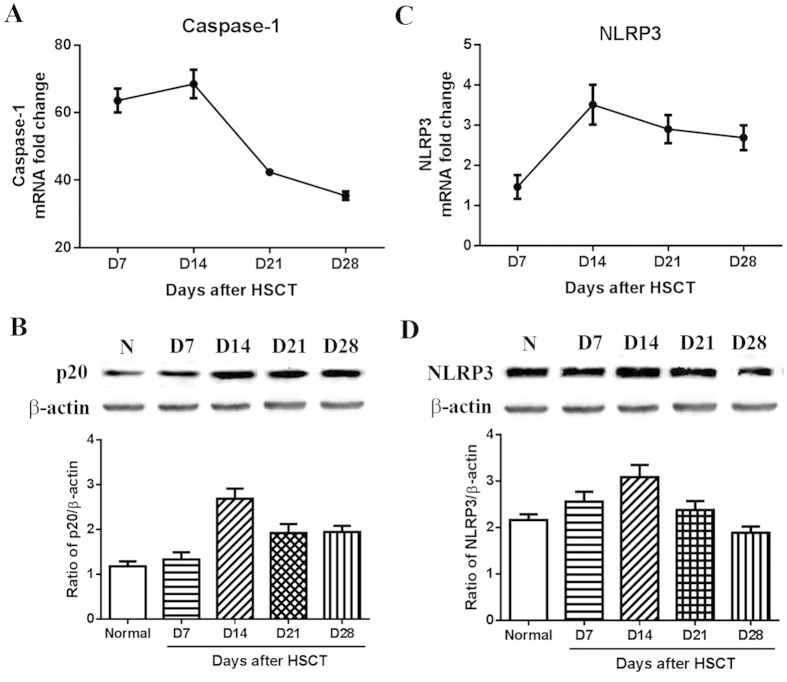
Expression of caspase-1 and NLRP3 in liver after HSCT. At indicated time points after HSCT, RNA and protein were isolated from liver for the measurement of mRNA and protein expression of caspase-1 and NLRP3 by quantitative real-time PCR and western blot respectively. Protein expressions of p20 and NPRP3 were quantified as a ratio to β-actin.

Increased secretion of IL-1β and IL-18 suggests the activity of caspase-1 might be enhanced as the maturation and production of these two cytokines depend on the activation of caspase-1. To confirm this, mRNA expression of caspase-1 and its active form (p20) was measured by quantitative PCR and western blot respectively (Fig. 3A,B). Consistent with the changes of the secretion of IL-1β and IL-18, expression of caspase-1 and p20 started to increase from d7 and achieved a maximum on d14 followed by a decline on d20, suggesting aberrant activation of caspase-1 in liver post HSCT.

Considering the regulation of inflammasome function by caspase-1, expression of most widely studied NLRP3 in liver on different time points post HSCT were measured. As seen in Fig. 3C,D, mRNA and protein level of NLRP3 increased reaching a maximum level on day 14 followed by a decline afterward, consistent with the changes of liver damage, inflammatory cell infiltration and secretion as well as caspase-1 activation, suggesting NLRP3 inflammasome activation might be responsible for the liver inflammatory injury after HSCT.

### Enhanced release of danger-associated molecular pattern molecules

**Figure 4:**
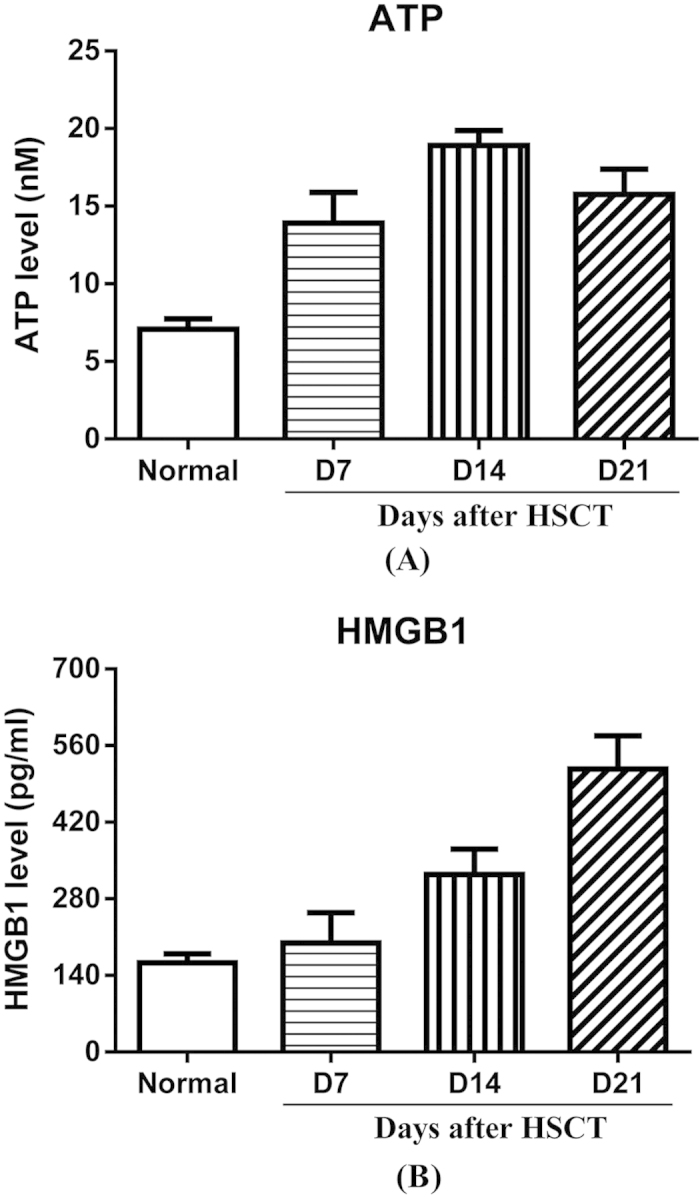
Level of ATP and HMGB1 in plasma. Plasma was isolated from mice at indicated time points after HSCT for analysis of release of ATP and HMGB1 by ELISA. Mice without any treatments were served as normal.

In light of the involvement of NLRP3 in liver inflammation after HSCT which was demonstrated as above, we sought to identify which molecule activated NLRP3. Danger-associated molecular pattern molecules (DAMPs) are molecules that are secreted, released or surface exposed by dying, stressed or injured tissues or cells and function as either adjuvant or danger signals for the immune system^19^. Previous *in vitro* and *in vivo* studies demonstrated ATP, as a class of DAMPs, to be a potent activator of NLRP3-dependent IL-1β release^20^,^21^,^22^. Therefore, we measured ATP level in plasma after HSCT. As seen in Fig. 4A, plasma ATP level increased after HSCT and reached a maximum on d14 followed by a decreased on the following days, which was consistent with the temporal changes of NLRP3 expression, indicating elevated ATP level might contribute to the activation of NLRP3 in liver post HSCT. In addition, we also measured the level of high-mobility group protein B1 (HMGB1), which is a pro-inflammatory agent whose secretion has been reported to be regulated by NLRP3^23^,^24^. Compared to normal, HMGB1 level started to dramatically increase from d14 to d21, which could be due to maximum activation of NLRP3 on d14 post HSCT. Taken together, these data demonstrated elevated ATP released after HSCT contributed to aberrant activation of NLRP3 and subsequent increased secretion of HMGB1.

### Ameliorated liver inflammation after inhibition of NLRP3

**Figure 5:**
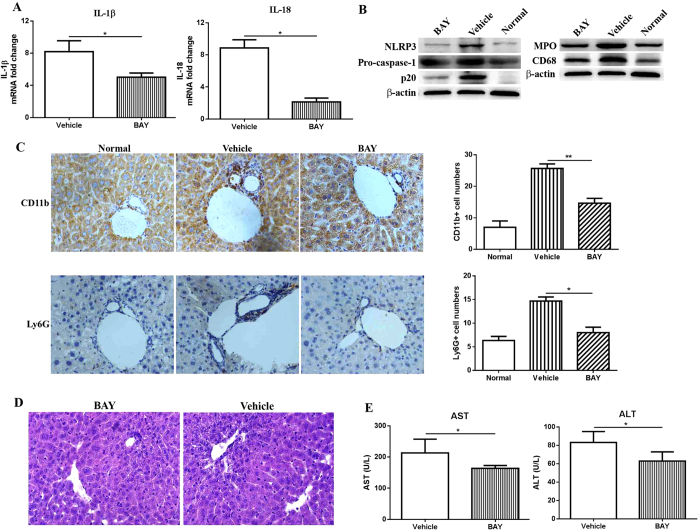
Secretion of IL-1β and IL-18, expression of NLRP3 and caspase-1, inflammatory cell infiltration and liver function in mice after treatment with BAY 11-7082. Mice receiving NLRP3 inhibitor (BAY) or vehicle were sacrificed on day 14 post HSCT and liver was obtained for analysis of the level of IL-1β and IL-18 in liver by quantitative real-time PCR (**A**). Expression of NLRP3 and caspase-1, inflammatory cells infiltration, such neutrophils and macrophages, were evaluated by western blot (**B**). In addition, immunohistochemical staining of CD11b and Ly6G expression in liver (**C**), H&E staining of liver (**D**) and liver function (AST and ALT level) measurement by automatic biochemistry analyzer (**D**) was also performed. A representative graph selected from 6 independent experiments at different time points was shown. ^**^P < 0.01, ^*^P < 0.05.

Given the association of NLRP3 expression with liver injury after HSCT, specific NLRP3 inhibitor, BAY 11-7082 was administered into mice through intraperitoneal injection after HSCT three times per week. Given the severest liver inflammation on day 14 post HSCT, mice receiving BAY 11-7082 were sacrificed on day 14 after transplantation for relevant parameters analysis. As seen in Fig. 5, lower expression of NLRP3 in liver was observed in treatment group (BAY) than that in control (Vehicle) as assessed by western blot. Meanwhile, mRNA level of IL-1β and IL-18 in liver were also significantly reduced after treatment with BAY 11-7082 (Fig. 5A), consistent with reduced caspase-1 activation as demonstrated by lower level of p20 (active form of caspase-1), caspase-1 and pro-caspase-1 (Fig. 5B). Taken together, these data demonstrated the dose of BAY 11-7082 used selectively inhibited NLRP3 inflammasome activation.

To evaluate inflammatory cell infiltration into liver after treatment with BAY 11-7082, proteins were isolated from liver for western blot analysis of the expression of CD68 and MPO. As seen in Fig. 5B, reduced expression of CD68 and MPO was observed in liver of mice treated with BAY, compared to vehicle group. Meanwhile, reduced infiltration of neutrophils were observed in liver of mice treated with BAY as demonstrated by decreased numbers of CD11b+ and Ly6G+ cells which were evaluated by immunohistochemistry (Fig. 5C). Consistent with this, H&E staining of liver also demonstrated less inflammatory cell infiltration after inhibition of NLRP3 function by BAY (Fig. 5D). In addition, improved liver function was also observed in mice treated with BAY as demonstrated by significantly lower level of AST and ALT compared to vehicle (P < 0.05) (Fig. 5E). Taken together, these data showed that improved liver function was observed after inhibition of NLRP3 which was possibly through reduced inflammatory cell infiltration to liver after HSCT.

## Discussion

Hematopoietic stem cell transplantation (HSCT) is potentially an effective strategy for the treatment of many hematological or non-hematological malignancies, such as leukemia, myeloma, solid tumors, or autoimmune diseases^25^,^26^,^27^. However, HSCT associated several complications were observed in some patients after transplantation, such as hepatic veno-occlusive disease (HVOD), influencing the treatment efficiency. Our previous study demonstrated that preparative regimen, such as busulfan combined with cyclosphomide (BU/CY) or total body irradiation (TBI), which was given to patients prior to HSCT for myeloablation, could cause severe liver inflammation, leading to liver sinusoid endothelial cell injury and subsequent the development of HVOD^9^. Given the critical role of NLRP3, the most studied inflammasome, in the regulation of inflammation, whether it is involved in liver inflammation after HSCT remains poorly understood. In this study, we demonstrated that increased NLRP3 expression and activation in liver was observed in mice after HSCT, with elevated release of ATP and HMGB1. Meanwhile, activation of caspase-1 and increased secretion of IL-1β and IL-18 were also found. Furthermore, inhibition of NLRP3 function by BAY 11-7082 ameliorated liver inflammation injury and improved liver function through reducing inflammatory cells infiltration.

The immune system is able to detect a wide range of insults against host including infections, tissue damage or metabolic stress^28^. The innate immune system possesses a large variety of germline encoded pattern recognition receptors (PRPs) through which it can detect such insults^29^. As a family member of intracellular immune receptors, the nucleotide-binding domain leucine-rich repeat–containing receptors (NLRs) are PRPs that initiate inflammatory response to a wide range of stimuli^28^. The NLR family consists of NLRP1, NLRP2, NLRP3, NLRP6, NLRC4, and NLRP12. Upon activation, the NLR family members are capable of forming multiprotein complexes named inflammasomes. The inflammasome is reported to play an important role in the regulation of innate immunity through participating in the production of the pro-inflammatory cytokines IL-1β and IL-18. These cytokines can initiate a wide variety of biological effects associated with infection, inflammation and autoimmune processes^30^. Consistent with this, our study showed an increased level of IL-1β and IL-18 in liver after HSCT, leading to severe liver inflammation. Previous study showed a new potential role for neutrophils in the pathogenesis of GVHD in both mice and humans after HSCT via tissue damage^31^. Consistent with this, our study demonstrated neutrophils might also play a role in liver inflammation after HSCT as demonstrated by increased infiltration of neutrophils into liver. Among NLR family members, NLRP3 is currently the most studied and fully characterized inflammasome, which responds to a large variety of chemical and physical stimuli. Given its critical role in the regulation of inflammation, its aberrant expression or function has been reported to be associated with various diseases, including infectious, autoinflammatory and autoimmune disorders, such as gout, type II diabetes, and cancer^32^. In addition, mutations of NLRP3 have been proposed to be a prognostic marker for the clinical outcomes of allogenic transplantation^33^. In this study, increased NLRP3 expression and caspase-1 activation in liver after HSCT was observed, consistent with liver pathology changes, indicating NLRP3 might be involved in the initiation of liver inflammation, which was further supported by that inhibition of NLRP3 ameliorated liver inflammation and improved liver function. However, considering BAY 11-7082, an NLRP3 selective inhibitor used in this study, being an inhibitor of cytokine-induced IκB-α phosphorylation, in the future, we plan to conduct further investigations on the role of NLRP3 in liver injury after HSCT using NLRP3 knockout mice.

Inflammasome assembly is unique in its induction by a variety of both exogenous and endogenous signals^34^. NLRP3 activation leads to assembly of NLRP3 inflammasome, which is composed of NLRP3, the adapter molecule ASC and pro-caspase-1^14^. This association and subsequent inflammasome activation result in caspase-1 activation, which cleaves pro-IL-1β and pro-IL-18 to their mature and secreted forms. The range of activation signals sensed by each inflammasome is distinct, but may include overlapping signals. NLRP3 is activated by a large variety of signals, such as environmental irritants (such as asbestos), endogenous danger signals, pathogen associated molecular patterns (PAMPs) (such as bacterial pore-forming toxins and the malaria parasite product haemozoin) and DAMPs (such as extracellular ATP)^35^. Previous studies demonstrated that release of PAMPs and DAMPs upon tissue damage after conditioning treatment triggered synthesis of pro-IL-1β and activation of NLRP3 inflammasome respectively, leading to an promoted inflammatory response which impacts the development and severity of graft versus host disease (GVHD)^36^,^37^. Consistent with the increased release of ATP upon tissue injury after conditioning treatment in HSCT mice model^37^, in this study we demonstrated that increased ATP level was found with reaching a maximum on d14 after transplantation conditioned with BU/CY. Although the mechanisms by how NLRP3 is oligomerized and activated in response to several cellular signals, such as lysosomes damage, increased generation of reactive oxygen species, and mitochondrial damage, are currently unclear, all of the proposed model agree with the critical role of cytoplasmic K^+^ concentration in inflammasome activation^35^. Meanwhile, K^+^ efflux from the cell should be factored into any proposed model for NLRP3 activation. As a potent activator of the NLRP3 inflammasome, ATP has the capacity to reduce intracellular K^+^ concentration by approximately 50%^38^, which is reported to be required for NLRP3 activation *in vitro*^39^. Consistent with being an activator for NLRP3 activation, ATP’s expression profile was similar to the level changes of NLRP3, suggesting the contribution of released ATP (might be the potential priming signal) to NLRP3 activation in liver after HSCT, leading to NRLP3 inflammasome assembly, caspase-1 activation and release of IL-1β and IL-18, which promoted an inflammatory response and infiltration of neutrophils and macrophages into liver, resulting in liver inflammatory injury, similarly to the pathogenesis and development of GVHD.

## Conclusions

In conclusion, our studies demonstrated increased NLRP3 and caspase-1 activation in liver together with elevated release of ATP and HMGB1 were observed after conditioned by BU/CY during HSCT and inhibition of NLRP3 activation reduced inflammatory cell infiltration, ameliorated liver inflammation and improved liver function, suggesting therapeutically targeting it might be beneficial in the prophylaxis and treatment of liver inflammation after HSCT.

## Materials and Methods

### Materials

BAY 11-7082 was purchased from Sigma-Aldrich (Lot No. B5556, St. Louis, MO, USA). Rabbit anti-mouse IL-1β antibody (Lot No. 12507) was from Cell Signaling TECHNOLOGY (Danvers, MA, USA). Rabbit polyclonal IL-18 antibody (Lot No. ab71495), rabbit anti-mouse myeloperoxidase (MPO) antibody (Lot No. ab139748), rabbit anti-mouse CD68 antibody (Lot No. ab53444), goat polyclonal anti-NLRP3 antibody (Lot No. ab4207) and rabbit monoclonal to CD11b antibody (Lot No. ab133357) were purchased from Abcam (Cambridge, MA). Rabbit polyclonal anti-caspase-1 antibody was from Biovision incorporated (Lot No. 3019-100, CA, USA). Rat anti-mouse endothelial cell monoclonal antibody (MECA-32) was purchased from BioLegend (Lot no. 120502, San Diego, USA). Rat anti-mouse Ly6G antibody was purchased from BD Biosciences (Lot No. 551459, San Jose, CA, USA)

### Animals and treatment

All animal care and experimental procedures were complied with Jiangsu Society for Animal Welfare for the Care and Use of Laboratory Animals and were approved by the Institutional Animal Care and Use Committee of Xuzhou Medical College. All animal care and experimental procedures followed ethical standards of animal use and were approved by Xuzhou Medical College. All experiments were carried out in accordance with the approved guidelines.

C57BL/6 (H-2Kb) (donor) and BALB/c (H-2Kd) (recipient) mice, aged 10–12 weeks and weighed 20–25 g, were purchased from SLAC LABORATORY ANIMAL, Shanghai, China. The mice were housed in sterilized cages at the Experimental Animal Center of Xuzhou Medical College.

HSCT mice model with BU/CY as preconditioning treatment was established as previously described^9^. For injection of NLRP3 inhibitor, 5 mg/kg (in 100 μl 10%DMSO) of BAY 11-7082 was administrated into each mouse (n = 6) via intraperitoneal injection after HSCT three times per week. At the same time, equal volume and concentration of DMSO was injected into mice after HSCT to serve as a control (vehicle).

### H&E staining and immunohistochemical staining

On d7, 14, 21 and 28 post HSCT, mice were sacrificed. Liver was isolated and then were fixed with formaldehyde solution, dehydrated, waxed, and sliced into 4  μm thickness by RM2126 microtome. After H&E staining, pathologic changes were evaluated by a light microscope.

Some liver slices were incubated with 3% H_2_O_2_ at room temperature and blocked with 5% goat serum. Then the slices were incubated with pan-endothelial cell monoclonal antibody (MECA-32) followed by incubation with secondary antibody which was HRP-conjugated rabbit anti-rat antibody. Color was developed with 3,3′-diaminobenzidine.

Regarding immunohistochemical staining of neutrophils infiltration after administration of NLRP3 selective inhibitor, anti-CD11b and anti-Ly6G antibodies were used.

### Mason staining

Mason staining of liver was performed using commercial trichrome stain (mason) kit (Lot No. D026, Nanjing Jiancheng Biotechnology Company Ltd, China) according to manufacturer’s instructions.

### Quantitative real-time PCR

Total RNA was isolated from liver of mice at indicated time points after HSCT with Trizol (Invitrogen, Massachusetts, USA) and cDNA was synthesized with a Moloney Murine Leukemia Virus Reverse Transcriptase (M-MLV) (Invitrogen, Massachusetts, USA). Expression of target genes was detected by quantitative PCR which was performed on LightCycler480 (Roche Diagnostics Ltd, Switzerland) by using Platinum SYBR Green qPCR Super Mix-UDG kit (Life Technologies, Massachusetts, USA) according to manufacturer’s instructions. β-actin was used as an internal control. Primers for target genes were shown in Table 2. Each sample was examined in triplicate. PCR product specificity was confirmed by a melting-curve analysis. The relative mRNA expression was determined by using 2^–ΔΔCt^ method.

### Western blot

Whole proteins were extracted from liver of mice after HSCT and were separated on 10% SDS-PAGE, followed by transferring to an NC membrane. The membranes were then blotted with rabbit anti-mouse IL-1β antibody, rabbit polyclonal IL-18 antibody, rabbit anti-mouse myeloperoxidase (MPO) or CD68 antibody, anti-NLRP3 antibody or rabbit polyclonal anti-caspase-1 antibody. Bound antibody was visualized using HRP-conjugated secondary antibody and enhanced chemiluminescence. Intensity of positive band was quantified using Image J software.

### Measurement of ATP and HMGB1

Levels of ATP and high-mobility group protein B1 (HMGB1) in plasma were measured by ELISA according to manufacturer’s instructions.

### Measurement of liver function

Level of alkaline phosphatase (ALT) and aspartate transaminase (AST) in plasma were measured by automatic biochemistry analyzer.

### Statistical analysis

Data was represented as Mean ± SE. Statistical significance between treatment group and vehicle on d14 was performed by two tailed, unpaired student t test using GraphPad Prism software (version 6.0). P < 0.05 was considered to be significantly different.

## Additional Information

**How to cite this article**: Qiao, J. *et al.* Busulfan and cyclosphamide induce liver inflammation through NLRP3 activation in mice after hematopoietic stem cell transplantation. *Sci. Rep.* **5**, 17828; doi: 10.1038/srep17828 (2015).

## Figures and Tables

**Table 1:** Pathology score of liver injury post HSCT.

Index	D7	D14	D21	D28
Endothelium injury in liver sinusoid or small hepatic veins	2	3	2	2
Hepatocyte necrosis	2/3	3	2	2
Subendothelial hemorrhage	0/1	2/3	0/1	1/2
Internal hemorrhage of hepatic sinusoid	1	2/3	1	2/3
Fibrosis of the central veins	1	2/3	1	2/3
Hepatic sinusoidal fibrosis	1	3	1	2
Inflammation of central veins	1/2	3	1/2	3
Total	7 ~ 9	18 ~ 21	9 ~ 11	11 ~ 13

**Table 2:** Quantitative PCR primers sequences.

Target genes	Primers sequence (5′-3′)	
	Forward	Reverse
IL-1β	CCTGAACTCAACTGTGAAATGC	GATGTGCTGCTGCGAGATT
IL-18	ATGGAGACCTGGAATCAGAC	TGTCAACGAAGAGAACTTGG
Caspase-1	ATCTTTCTCCGAGGGTTGG	AAGTCTTGTGCTCTGGGCAG
NLRP3	AAGAAGAGAGGAGAGGAGGTCG	CGGTTGGTGCTTAGACTTGA
β-actin	CTGAGAGGGAAATCGTGCGT	AACCGCTCGTTGCCAATAGT
